# Supplementary material for: Fine-Scale Crossover Rate Variation on the Caenorhabditis elegans X Chromosome
Source: G3 (Bethesda). 2016 Apr 15;6(6):1767–76. doi: 10.1534/g3.116.028001 (PMC4889672; doi:10.1534/g3.116.028001)
Supplement: Supplemental Material [file supp_6_6_1767__index.html]

Fine-Scale Crossover Rate Variation on the Caenorhabditis elegans X Chromosome — Supplemental Material 

# Fine-Scale Crossover Rate Variation on the *Caenorhabditis elegans* X Chromosome

## Supplemental Material for Bernstein and Rockman, 2016

**Files in this Data Supplement:**

- Figure S1 - Genotyping scheme. (.pdf, 468 KB)
- Figure S2 - Small sequence motif permutation test pipeline. (.pdf, 449 KB)
- Table S1 - Data from modENCODE. (.pdf, 72 KB)
- Table S2 - Association of histone modifications with crossover distribution. (.pdf, 71 KB)
- Table S3 - Explanatory power of small motifs after removing CB4856 deletions. (.pdf, 76 KB)
- File S1 - crossovers\_full.csv. (.csv, 6 KB)
- File S2 - crossovers\_25kb.csv. (.csv, 2 KB)
- File S3 - omitted\_intervals.csv. (.csv, 1 KB)
- File S4 - alt\_crossovers\_25kb.csv. (.csv, 2 KB)
- File S5 - motif\_counter.R. (.zip, 3 KB)
- File S6 - focal\_interval\_DNA.txt. (.txt, 1448 KB)
- File S7 - deviance\_sim.R. (.zip, 2 KB)
- File S8 - motif\_perms.R. (.zip, 2 KB)
