# Supplementary material for: Fine-Scale Crossover Rate Variation on the Caenorhabditis elegans X Chromosome
Source: G3 (Bethesda). 2016 Apr 15;6(6):1767–76. doi: 10.1534/g3.116.028001 (PMC4889672; doi:10.1534/g3.116.028001)
Supplement: Supplemental Material [file supp_g3.116.028001_FigureS1.pdf]

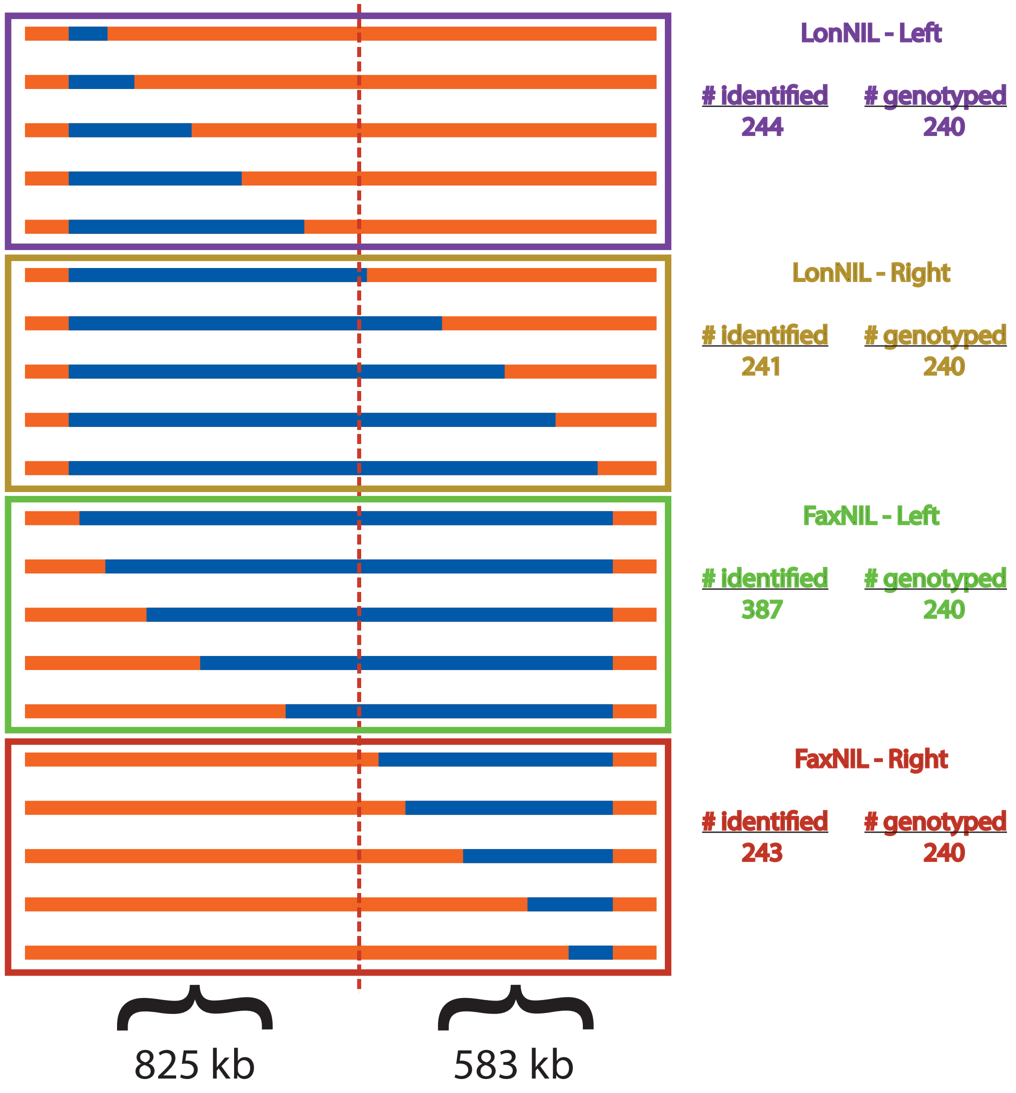

Figure S1: Genotyping scheme. We classified recombinant sub-NILs into four groups dependent on parental origin (LonNIL or FaxNIL) and location of the crossover (Left or Right) inferred from indel (vertical dashed line) genotyping. Although we recovered unequal numbers of each class, we submitted 240 of each for Illumina GoldenGate genotyping. This unequal representation combined with the unequal physical distances comprising the left and right sides of the indel necessitates a covariate in our generalized linear models to avoid spurious associations.
