# Supplementary figures and images for: Fine-Scale Crossover Rate Variation on the Caenorhabditis elegans X Chromosome
Source: G3 (Bethesda). 2016 Apr 15;6(6):1767–76. doi: 10.1534/g3.116.028001 (PMC4889672; doi:10.1534/g3.116.028001)

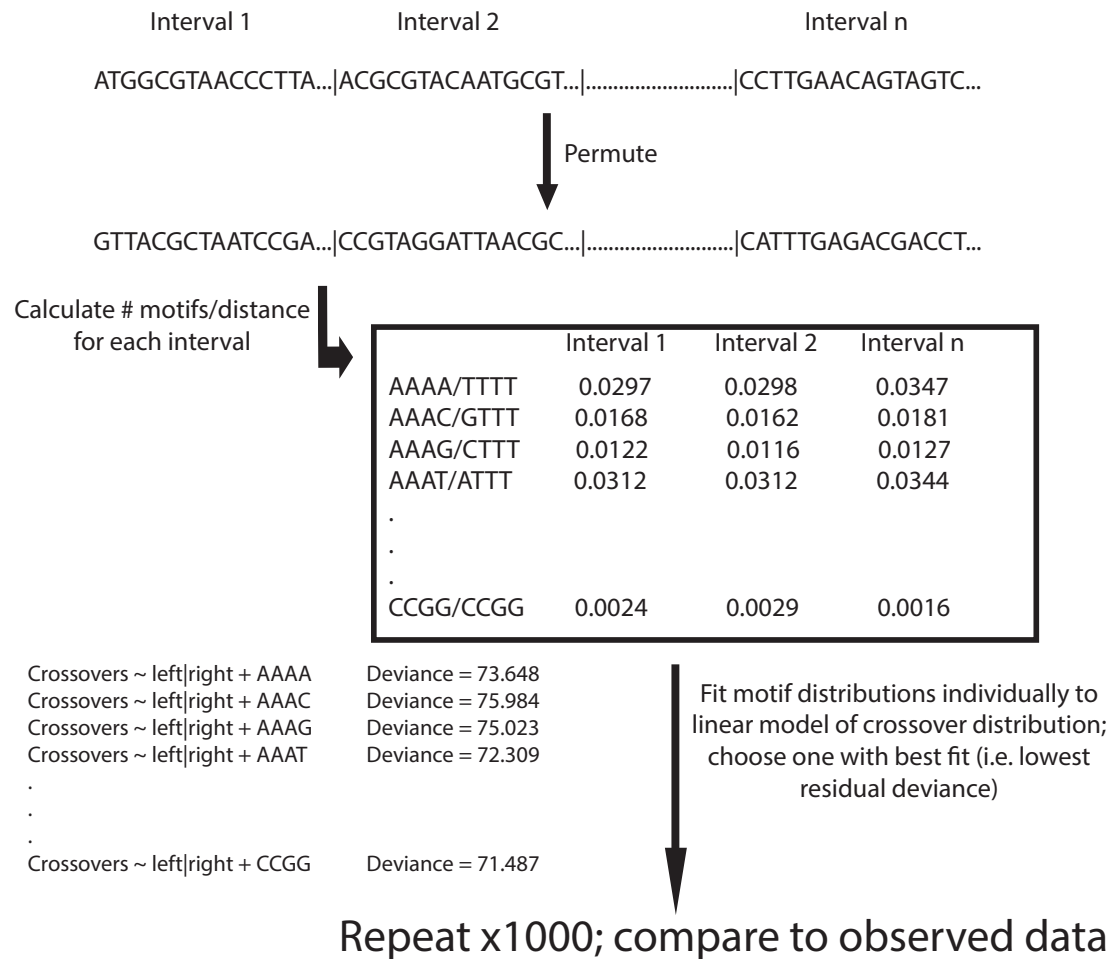

**Figure S2:** Small sequence motif permutation test pipeline.

Supplement: Supplemental Material [file supp_g3.116.028001_FigureS2.pdf]
