# Supplementary material for: Fine-Scale Crossover Rate Variation on the Caenorhabditis elegans X Chromosome
Source: G3 (Bethesda). 2016 Apr 15;6(6):1767–76. doi: 10.1534/g3.116.028001 (PMC4889672; doi:10.1534/g3.116.028001)
Supplement: Supplemental Material [file supp_g3.116.028001_TableS1.pdf]

Table S1: Data from modENCODE

Mark	Track	DCCid
H3K4me1	AB8895_H3K4ME1733246_N2_EEMB	modENCODE_2726
H3K4me2	WA30834809_H3K4ME2_N2_EEMB	modENCODE_3198
H3K4me3	WA30534819_H3K4ME3_N2_EEMB	modENCODE_3197
H3K9me1	AB9045_H3K9ME1291918_N2_EEMB	modENCODE_2646
H3K9me2	HK00008_H3K9ME26D11_N2_EEMB	modENCODE_2444
H3K9me3	AB8898_H3K9ME3339901_N2_EEMB	modENCODE_2339
H3K27ac	AB4729_H3K27AC361571_N2_EEMB	modENCODE_2740
H3K27me1	UP07448_H3K27ME124439_N2_EEMB	modENCODE_3179
H3K27me3	HK00013_H3K27ME3:1E7_N2_EEMB	modENCODE_3171
H3K36me1	AB9048_H3K36ME1206009_N2_EEMB	modENCODE_2604
H3K36me2	AB9049_H3K36ME2608457_N2_EEMB	modENCODE_2338
H3K36me3	HK00001_H3K36ME3_13C9_N2_EEMB_s1	modENCODE_3553
H3K79me2	AB3594_H3K79ME2346021_N2_EEMB	modENCODE_2442
H3K79me3	AB2621_H3K79ME3361576_N2_EEMB	modENCODE_2443
H4K8ac	AB15823_H4K8AC487128_N2_EEMB	modENCODE_3181
H4K16ac	MP07329_H4K16ACDAM1612187_N2_EEMB	modENCODE_3182
H4K20me1	DISN147_H4K20ME1_001_N2_EEMB	modENCODE_3434
IgG	AB46540_NIGG_N2_MXEMB	modENCODE_3153
LEM2	SDQ3891_LEM2_N2_MXEMB	modENCODE_2729
Mononucleosome	Mononucleosomes_N2_MXEMB	modENCODE_2763
