# Supplementary material for: Fine-Scale Crossover Rate Variation on the Caenorhabditis elegans X Chromosome
Source: G3 (Bethesda). 2016 Apr 15;6(6):1767–76. doi: 10.1534/g3.116.028001 (PMC4889672; doi:10.1534/g3.116.028001)
Supplement: Supplemental Material [file supp_g3.116.028001_TableS2.pdf]

Table S2: Association of histone modifications with crossover distribution

Modification	Coefficient	Deviance Explained	p
H3K4me1	-0.21	3.4%	0.113
H3K4me2	-0.13	4.7%	0.064
H3K4me3	-0.22	7.6%	**0.019**
H3K9me1	-0.38	14.6%	**0.001**
H3K9me2	-0.58	14.9%	**0.001**
H3K9me3	-0.20	2.0%	0.229
H3K27ac	-0.16	4.5%	0.070
H3K27me1	-0.16	11.0%	**0.005**
H3K27me3	0.18	0.7%	0.467
H3K36me1	-0.38	12.4%	**0.003**
H3K36me2	-0.27	14.3%	**0.002**
H3K36me3	-0.31	9.4%	**0.011**
H3K79me2	-0.18	11.7%	**0.004**
H3K79me3	-0.18	12.8%	**0.003**
H4K8ac	-0.05	0.1%	0.770
H4K16ac	-0.19	3.7%	0.102
H4K20me1	-0.29	10.4%	**0.007**
