# Supplementary material for: Fine-Scale Crossover Rate Variation on the Caenorhabditis elegans X Chromosome
Source: G3 (Bethesda). 2016 Apr 15;6(6):1767–76. doi: 10.1534/g3.116.028001 (PMC4889672; doi:10.1534/g3.116.028001)
Supplement: Supplemental Material [file supp_g3.116.028001_TableS3.pdf]

Table S3: Explanatory power of small motifs after removing CB4856 deletions

**Sequence(s)**	**Resolution**	**N2 sequence p-value**	**≥100 bp deletion p-value**	**≥50 bp deletion p-value**	**≥20 bp deletion p-value**
AACA	Full	**0.004**	**0.002**	**0.004**	**0.002**
GGATAG	25 kb	**0.040**	**0.037**	**0.022**	**0.046**

Significance reported in Table 3 is listed here as "N2 sequence p-value." Subsequent columns indicate the lengths of CB4856 deletions (Thompson et al., 2015) removed from the N2 sequence. Permutation tests were performed as described in the Materials and Methods and Figure S2 on edited DNA sequence.
